# Rationalizing the Influence of Solvent on the Nucleation of Griseofulvin through Classical and Nonclassical Pathways

**DOI:** 10.1021/acs.cgd.5c00206

**Published:** 2025-06-03

**Authors:** Mariana O. Diniz, Harsh Barua, Jennifer Cookman, Michael Svärd, Åke Rasmuson, Sarah P. Hudson

**Affiliations:** † SSPC the Research Ireland Centre for Pharmaceuticals, Department of Chemical Sciences, and Bernal Institute, 8808 University of Limerick, Limerick V94 T9PX, Ireland; ‡ Department of Chemical Engineering, KTH Royal Institute of Technology, Stockholm SE-10044, Sweden

## Abstract

The effect of solvent on active pharmaceutical ingredient (API) nucleation behavior is system-dependent. A better understanding of the role of the solvent in nucleation could help predict and control crystallization. In this work, induction time experiments, spectroscopic analysis, and dynamic light scattering were used to explore the influence of solvent on the polymorphic landscape and the nucleation behavior of griseofulvin (GSF), a medium-sized, flexible, model API. Based on a total of 2960 induction time experiments, the relative ease of nucleation was characterized in three solvents commonly used in the pharmaceutical industry: methanol (MeOH), acetonitrile (ACN), and *n*-butyl acetate (nBuAc). GSF crystallized as stable Form I in MeOH and as solvated forms in both ACN and nBuAc. GSF nucleated most easily in ACN, followed by nBuAc, while nucleation was most difficult in MeOH. This order was found to correlate with increasing interfacial energy, which was found to be lower in ACN, intermediate in nBuAc, and higher in MeOH, based on a classical evaluation. However, in contrast to classical nucleation theory, which suggests that higher nucleation rates are associated with larger pre-exponential factors, the pre-exponential factor was found to be highest in MeOH, while it remained comparable in ACN and nBuAc. An analysis of the GSF solutions used in the nucleation studies confirmed the presence of mesoscale clusters in ACN and in nBuAc, but not in MeOH. The size and concentration of mesoscale clusters in ACN solution were higher than those in nBuAc, which could explain the higher nucleation rate observed in ACN if the nonclassical nucleation pathway is considered for these solvents.

## Graphical abstract

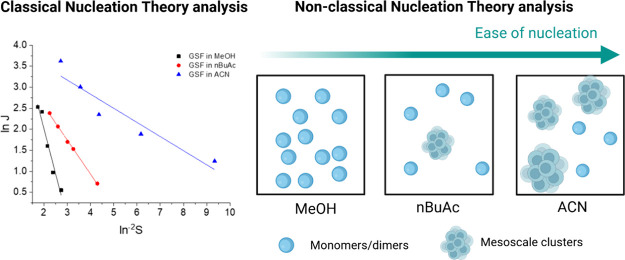

## Introduction

Crystallization is an important purification, separation, and formulation process for the pharmaceutical industry. It impacts the resulting purity, solid-state, crystal habit, and particle size of the active pharmaceutical ingredient (API). These properties can affect the solubility, and therefore the bioavailability^1,2^ as well as important powder properties such as flowability, compatibility, and filtration behavior, which in turn determine the manufacturability of the drug.^3,4^ Crystallization is a two-step process involving nucleation, where a stable nucleus separates from solution, and crystal growth, where the nucleus grows into the final crystal.^5^ Nucleation is a poorly understood phenomenon due to its stochastic nature and difficulty in detection due to the small size of the formed nuclei and short time scale of formation.^6^ Crystallization from solution occurs when supersaturation is achieved,^7,8^ which means the solute concentration exceeds its equilibrium solubility in solution.^7^ Therefore, determination of the solubility of the API in solution is normally the first step in the design of a crystallization process.

The nucleation rate, expressed as the number of new nuclei formed per unit time and volume, serves as a quantitative measure of the difficulty of nucleation. At identical supersaturation levels, a higher nucleation rate indicates that nucleation is kinetically less hindered, meaning that a lower supersaturation (or driving force) is required for nucleation to occur within a given time frame.^9–12^ Nucleation kinetics can be determined through induction time experiments, where the crystallization temperature is kept constant and the time until the first crystals are detected is recorded;^9,12,21–27,13–20^ or by metastable zone width experiments, where the cooling rate is kept constant and the temperature at which nucleation occurs is recorded.^28–32^ Both methods allow the use of the classical nucleation theory (CNT) equation, see the classical nucleation theory- theoretical description section, to estimate the kinetic factor (pre-exponential-A), related to the rate of attachment of molecules to the critical nucleus and the diffusivity, and the thermodynamic factor (interfacial energy-γ), related to the interactions at the solid–liquid interface such as hydrogen bonding and solvation.^10,32,33^ The induction time (*t*_ind_) is the sum of the relaxation time required for the system to reach the quasi-steady distribution of molecule clusters; the time to form a stable nucleus (nucleation time, *t*_nuc_); and the time for nucleus growth to a detectable size (*t*_g_).^33^ The relaxation time depends on the viscosity and diffusivity of the system, the nucleation time depends on the supersaturation, and the growth time depends on the size at which nuclei are detectable and on the growth rate applicable at this stage.^33^ In solutions of moderate viscosity and supersaturation, the relaxation time can be neglected.^33^ Therefore, in most cases, the nucleation time is given by *t*_nuc_ = *t*_ind_ – *t*_g_.

The CNT was the first model developed to try to explain the nucleation pathway.^34,35^ It postulates that gradual attachment and detachment of monomer growth units to a cluster under favorable energy conditions eventually result in a nucleation event, the creation of a stable nucleus,^32^ which can subsequently grow to reach the final crystal size.^32^ According to the CNT, the nucleation rate increases with an increase in the pre-exponential factor and with a decrease in interfacial energy.^33^ The influence of the solvent on nucleation has previously been evaluated for many APIs and has been shown to be very system-dependent. In the case of flufenamic acid, it was shown that nucleation becomes easier with an increase in pre-exponential factor and a decrease in interfacial energy, aligning with the CNT.^25^ For fenoxycarb,^36^ benzocaine,^11^ phenacetin,^37^ 3,5-dinitrobenzoic acid,^18^ tolfenamic acid,^38^ tolbutamide,^29^ and ethyl and propyl paraben,^39^ the nucleation rate was shown to increase with a decrease in interfacial energy. For salicylamide,^14^ the nucleation rate increased with increased pre-exponential factor and interfacial energy.

Nonclassical nucleation models were later developed, specifically accounting for the intermediate stages before reaching a thermodynamically stable phase, which are not described in the CNT.^6,40^ The two-step nucleation pathway was developed to explain density fluctuations in protein crystallization preceding nucleation, thought to occur inside intermediate, liquid-like clusters.^41^ The prenucleation cluster (PNC) pathway was proposed to explain intermediate species, postulated to be thermodynamically stable, highly dynamic, and possibly containing structural motifs related to the nucleating phase.^42^ The intermediate species, widely detected and often assumed to be instrumental for nucleation, have frequently been reported to lack long-range order and a distinct phase boundary.^40^ In the case of organic molecules, the term “mesoscale clusters” has been used for aggregates in solution with sizes in the approximate range from 10 to 1000 nm, generally held to be composed of both solute and solvent molecules.^40,43^ Mesoscale clusters have been detected by techniques such as dynamic light scattering (DLS),^44–49^ nanoparticle tracking analysis (NTA),^45,46,48,49^ small-angle scattering of X-rays (SAXS)^45,46,48^ or neutrons (SANS),^50,51^ cryogenic transmission electron microscopy (cryo-TEM)^47,49,52,53^ and liquid phase transmission electron microscopy (LPTEM).^54^ The presence of mesoscale clusters enhanced the nucleation rate of glycine in aqueous solution,^46^ while larger cluster sizes or higher cluster number concentrations further increased the nucleation rate for 2-cyano-40-methylbiphenyl, salicylamide, fenoxycarb, and salicylic acid in organic solvents.^45,55,56^

The (albeit few) studies reported thus far highlight that further investigation into the system-dependent influence of the solvent on mesoscale clustering and nucleation is required. In this work, the impact of three different solvents: methanol (MeOH; polar protic), acetonitrile (ACN; polar aprotic), and *n*-butyl acetate (nBuAc; polar aprotic), on the nucleation of griseofulvin, a medium-sized, flexible, and polymorphic model API, was investigated. GSF (molecular weight of 352.8 g/mol) is an antifungal antibiotic used to treat dermatophyte infections.^57,58^ It is prone to polymorphism and solvate formation, having 6 polymorphs^59–62^ and 13 solvated forms^62–67^ reported in the Cambridge Structural Database (CSD).^68^ The chemical structures of GSF, MeOH, ACN, and nBuAc are presented in Figure 1. MeOH and ACN are classified according to the International Conference on Harmonization of Technical Requirements for Registration of Pharmaceuticals for Human Use (ICH) guideline as class 2 solvents, which means they present low severity and reversible toxicity, and their use should be limited. nBuAc is a class 3 solvent, which means it presents low toxicity and should be used where practical.^69^ GSF typically nucleates as solvated forms in ACN^66^ and in nBuAc^62^ and as the stable Form I in MeOH.^59^ The influence of the solid phase on the solubility of GSF was investigated by determining GSF solvate solubilities and comparing to the solubility of Form I as reported by Zhao et al.^77^ The effect of solubility on nucleation was also analyzed, given that the GSF-ACN solubility in ACN is ten times higher than that of GSF-nBuAc in nBuAc and of GSF Form I in MeOH. Experimentally measured nucleation kinetics are evaluated with the use of classical and nonclassical nucleation theories. Finally, the presence of mesoscale clusters was investigated in order to present a comprehensive view of the feasible nucleation pathways of GSF in different solvents.

**Figure 1:**
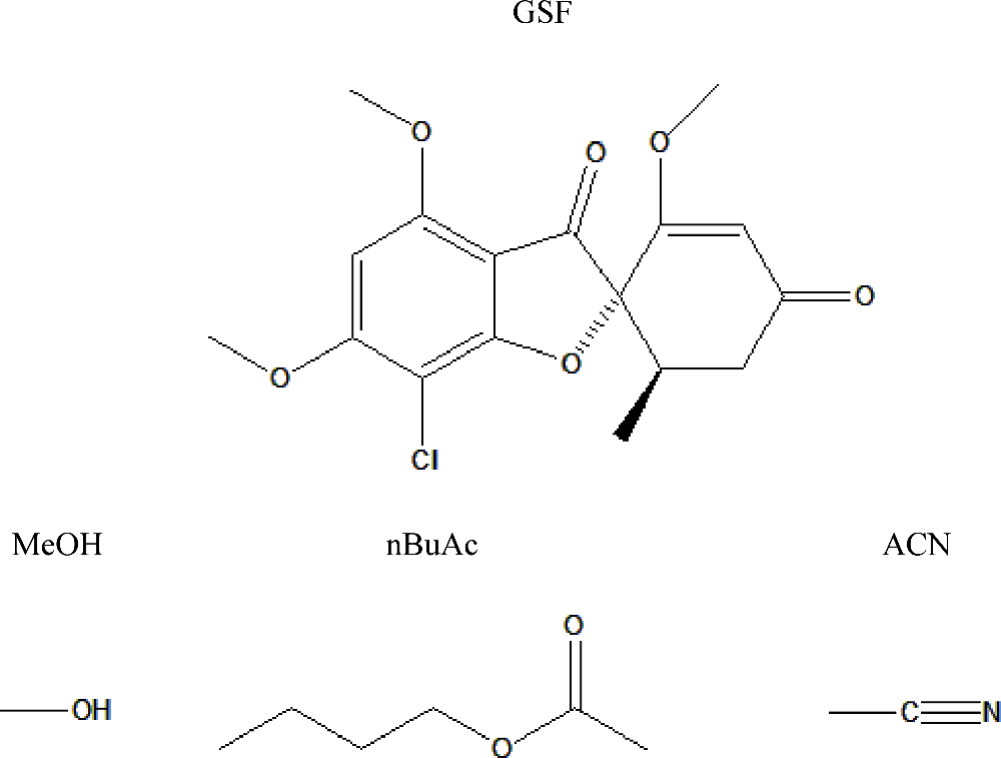
Chemical structures of GSF, MeOH, nBuAc, and ACN.

## Experimental Section

### Materials

Griseofulvin (GSF) Form I (98%) was obtained from Baoji Guokang Bio-Technology Co. (China), and the solvents methanol (MeOH; 99.8%), *n*-butyl acetate (nBuAc; 99.0%), and acetonitrile (ACN; 99.9%) were obtained from Fisher Scientific. All chemicals were used without further purification.

### Induction Time Measurements

Induction times for crystal nucleation of GSF were measured at 283 K in three solvents: MeOH, nBuAc, and ACN. Two digitally controlled, stirred thermostatic water baths and circulators (Grant GR150-S26) fitted with serial submersible magnetic stirrer plates (2Mag) with 60 points and a submersible water pump (1400 L h^–1^) were employed to monitor the nucleation experiments. The set temperature was validated by a digital thermometer (VWR traceable thermometer).

Stock solutions were prepared in a Duran bottle by weighing in the appropriate amounts of solid (Mettler Toledo AX504 analytical balance; ±0.0001 g) and solvent (Mettler Toledo XS6002S precision balance; ±0.001 g). Solutions were prepared at five concentrations for each solvent within the respective range: 5.9–6.9 g kg^–1^ in MeOH, 9.1–11.1 g kg^–1^ in nBuAc, and 47.4–62.7 g kg^–1^ in ACN. The resulting solutions were stirred at 500 rpm using polytetrafluoroethylene (PTFE) magnetic stirring bars (Fisherbrand, 11 × 25 mm) and maintained at the dissolution temperature for 72 h. The dissolution temperature was 313 K for MeOH and ACN, and it was 333 K for nBuAc.

The resulting solutions were then filtered using preheated syringes and filters. Aliquots of 20 mL were transferred to 20 preheated glass vials (Fisherbrand, screw neck clear glass, 30 mL, 27.5 × 72.5 mm) containing a magnetic stirrer bar (Merck, PTFE coated, 1/2 × 1/8 in) using suitable syringes (SLS, 3-part polypropylene, 50 mL for MeOH and ACN, and Discardit, 2-part polypropylene, 20 mL for nBuAc) and filters (Fisherbrand, 0.2 μm, PTFE). The vials were sealed using screw-cork caps (LLG, 24 mm, polypropylene, septa butyl/PTFE) and further sealed using parafilm to prevent solvent evaporation. The vials were kept at the dissolution temperature and stirred at 1200 rpm for 24 h.

Supersaturated solutions were obtained by cooling the solutions to a temperature (designated nucleation temperature), where the solubility would be below the solution concentration. Supersaturation ratios (*S*) were calculated as *S* = *x*/*x*_eq_, where *x* is the solute concentration in the solution in mole fraction and *x*_eq_ is the solute crystallizing equilibrium solubility at the nucleation temperature, in mole fractions. Supersaturation ratios with respect to crystallizing form (1.83–2.14 in MeOH, 1.62–1.95 in nBuAc, and 1.39–1.83 in ACN) were achieved by transferring the vials to a water bath at the nucleation temperature of 283 K. The time lag for the temperature to reach the nucleation temperature was monitored by a precision digital thermometer (VWR traceable, resolution 0.001 K, precision ± 0.05 K). In all cases, the temperature was reached within 2 min, and no vial had visibly nucleated during the cooling stage. The visible onset of nucleation was recorded by using a high-definition resolution video camera (SONY HDR-XR520). The induction time was established as the time elapsed following the placement of the clear solutions in the nucleation temperature bath until the first visible cloudiness was detected, corresponding to the detection of the first crystals. The progression from clear solution via the onset of visible cloudiness to complete cloudiness was always rapid, and the estimated uncertainty associated with the induction time observations is in all cases below 10 s. The vials were kept at the nucleation temperature for 8 h, after which the crystals were redissolved at the dissolution temperature for 16 h. The stirring rate was kept at 1200 rpm in all stages. The cycle was repeated 4 times for a total of 80 experiments per solution. For MeOH and nBuAc, a minimum of two fresh solutions for each of the five evaluated concentrations were prepared, resulting in a total of at least 160 experiments at each supersaturation level. For ACN, which presented lower variability in *t*_ind_, two fresh solutions were prepared for three concentrations and one for the remaining concentrations. This resulted in a total of 2960 experiments. The experimental data sets were evaluated for statistical similarity using the Kolmogorov–Smirnov (K–S) test at a significance level of 0.05. This test compares the empirical cumulative distribution functions (CDFs) of the data sets, calculating the maximum absolute difference between them to determine the p-value. Data sets with p-values exceeding the significance level were deemed statistically similar and subsequently combined. Conversely, data sets with p-values below 0.05 were considered statistically different and were analyzed separately.^70,71^

The median induction time (*t*_50_) was extracted directly from the experimental data as the time in which 50% of the vials were nucleated, and the growth time (*t*_g_) was determined as the first induction time point.

### Solid-Phase Characterization

The samples of GSF solids nucleated in MeOH, nBuAc, and ACN were analyzed by PXRD. Suspensions of GSF crystals were collected after the last nucleation cycle and filtered using 0.22 μm poly­(vinylidene fluoride) (PVDF) membrane filters. A thin layer of the resulting powder was placed on the zero background disks. PXRD data were collected in reflection mode with an Empyrean diffractometer (PANalytical, Phillips) equipped with CuKα radiation (λ = 1.5406 Å) operating at 45 kV and 40 mA at room temperature. Samples were scanned between 2θ values of 5° and 45° at a step size of 0.01313° s^–1^, 18.87 s per step.

### Solubility at 283 K

The GSF Form I and GSF-ACN, and GSF-nBuAc solvate solubilities at 283 K were measured by a gravimetric method using a five-decimal precision balance (Mettler Toledo, Model XP205). In a vial (Fisher, Screw Neck clear glass, 30 mL, 27.5 × 72.5 mm), excess GSF Form I was added to the respective solvent for measuring the solubility of GSF Form I. The slurry of the solvate after nucleation was used for measuring the solubility of the GSF solvate. The suspension was stirred at 1200 rpm for 24 h at 283 K to reach solid–liquid equilibrium. The stirring was turned off for 1 h to let the solids in the suspension settle. The solid phase present after equilibration was analyzed by PXRD as described above. Aliquots of 5 mL of the solution were filtered using 0.2 μm PTFE filters into preweighted vials (*m*_vial+cap_) (Fisherbrand, soda lime glass, push-in closure, 16 mL, 25 × 50 mm). Immediately after filling, the evaporation vials were capped and weighed (*m*_vial+cap+solution_). The vials were then left open inside a fume hood at room temperature (293–298 K) to let the solvent evaporate. The vials containing only the solids after the completion of solvent evaporation, confirmed by constant weight, were finally weighed (*m*_vial+cap+solid_). To assess reproducibility, three samples of each condition were analyzed. The mass ratio solubility (C*) was calculated according to eq 1:

$$C^{*}\left(\frac{g\;\mathrm{GSF}}{g\;\mathrm{solvent}}\right)=\frac{m_{\mathrm{vial}+\mathrm{cap}+\mathrm{solid}}-m_{\mathrm{vial}+\mathrm{cap}}}{m_{\mathrm{vial}+\mathrm{cap}+\mathrm{solution}}-m_{\mathrm{vial}+\mathrm{cap}+\mathrm{solid}}}$$ 1

The mole fraction solubility (*x*_eq_) was calculated from the mass ratio solubility (C*) as described in eq 2:

$$x_{\mathrm{eq}}=\frac{C^{*}M_{2}}{C^{*}M_{2}+M_{1}}$$ 2

where *M*_1_ and *M*_2_ stand for the molecular weights (g mol^–1^) of the solute and solvent, respectively.

### Mesoscale Cluster Analysis

GSF solutions were analyzed by dynamic light scattering (DLS) using a Malvern Zetasizer ZSP Nano instrument equipped with a temperature controller and a red laser (λ = 632.8 nm). Malvern Zetasizer software v 7.11 was used to analyze the scattering data. The samples were prepared following the induction time analysis procedure at a concentration that resulted in a supersaturation level of *S* = 1.83 upon cooling to 283 K. Solutions were analyzed at *t* = 0 h, corresponding to the start of the first nucleation run. The analysis was undertaken with two independent samples from two fresh solutions for each condition to assess the reproducibility. Samples of pure solvents were also analyzed to be used as a blank comparison. Aliquots of 2 mL of solution were added to a quartz cuvette. Each measurement comprised eight runs, with each run consisting of ten subruns, conducted at 283 K. Measurements from the final three runs were retained for analysis, while the initial runs were considered necessary for equilibrating the sample to the measurement temperature. No nucleation was observed during the analysis. The measurements were conducted in backscattering mode at an angle of 173°. Distribution analysis was used for the deconvolution of the correlation graph, which is further utilized to calculate the solvodynamic diameter (*D*_s_) via the Stokes–Einstein equation eq 3:

$$D_{s}=2R_{s}=\frac{2k_{B}T}{6\pi \eta D}$$ 3

where *R*_s_ is the solvodynamic radius, *k*_B_ is the Boltzmann constant, *T* is the temperature in Kelvin, η is the dynamic viscosity of the solvent, and *D* is the diffusion coefficient. The derived count rate, expressed in counts per second and indicative of the scattered intensity (the number of scattered photons reaching the detector), was monitored for each measurement.^72^ The solvent viscosity (Table S2) was utilized for the calculation of *D*_s_, as it was assumed that the concentration of GSF was too low to affect viscosity significantly.

### Classical Nucleation Theory: Theoretical Description

According to CNT, the energy barrier to nucleation, also known as nucleation work, of a spherical critical nucleus of radius *r* is given by^33^

$$\Delta G(r{)}^{*}=\frac{16\pi \gamma^{3}\upsilon^{2}}{3(k_{B}T\ln\;S{)}^{2}}=\frac{B}{k_{B}T\ln^{2}\;S}$$ 4

where γ is the solid–liquid interfacial energy (J m^–2^), υ is the volume of one solute molecule (m^3^), *k*_B_ is the Boltzmann constant, *T* is the absolute temperature (K), *S* is the supersaturation ratio, and *B* is the thermodynamic factor. The radius of a spherical critical nucleus at a given supersaturation is expressed as^33^

$$r^{*}=\frac{2\gamma \upsilon }{k_{B}T\ln\;S}$$ 5

The number of molecules in the critical nucleus (*N*_c_) is given by^39^

$$N_{c}=\frac{4\pi r^{*3}}{3\upsilon }$$ 6

The nucleation rate (*J*) is the number of stable nuclei formed in the system per unit of volume (*V*) and time (*t*).^73^ The nucleation rate can be expressed in the form of the Arrhenius reaction velocity:^33^

$$J=A\;\exp\left[-\frac{B}{\ln^{2}\;S}\right]$$ 7

where *A* is the kinetic pre-exponential factor derived from the Arrhenius equation (m^–3^ s^–1^) and *B* is the thermodynamic factor that is given by eq 8:

$$B=\frac{16\pi \gamma^{3}\upsilon^{2}}{3{k_{B}}^{3}T^{3}}$$ 8

The nucleation rate can also be determined by the fitting of the induction time distribution with the single nucleus mechanism (SNM) equation (eq 9).^74,75^

$$P(t)=1-\exp\{-JV(t-t_{g})\}$$ 9

The coefficient of variation (CV) describes the spread of nucleation time and is given by the ratio between the standard deviation and the average as presented in eq 10:^76^

$$\mathrm{CV}=\frac{s\tan\mathrm{dard}\;\mathrm{deviation}\;\mathrm{of}\;\mathrm{the}\;\mathrm{induction}\;\mathrm{time}\;\mathrm{distribution}}{\mathrm{average}\;\mathrm{of}\;\mathrm{induction}\;\mathrm{time}\;\mathrm{distribution}}$$ 10

## Results and Discussion

### Solid-Phase Characterization and Solid-Liquid Solubility

PXRD analysis indicated that GSF nucleated as Form I in MeOH (CSD reference code GRISFL), as a GSF-nBuAc solvate in nBuAc (CSD reference code QIZDIQ), and as a GSF-ACN solvate in ACN (CSD reference code PINMOQ) (Supporting Information, Figures S1–S3). The solubilities of GSF-ACN in ACN and GSF-nBuAc in nBuAc were determined at a nucleation temperature, *t*_nuc_, of 283 K, as shown in Table 1. The order of solubility in mole fraction of GSF Form I at 283 K determined in this work, starting with Form I, agreed with that determined by Zhao et al. as presented in Table 2. The solubility of both solvated forms was higher than the solubility of Form I in the respective solvent. This agreed with the trend of higher solubility for solvates compared to the desolvated form, as reported in the literature.^77,78^ These results also confirmed the trend of solubility previously reported for GSF Form I,^77^ in which the solubility is higher in ACN, followed by nBuAc and then MeOH. The resulting PXRD analysis of the solid state after equilibration starting from the slurry of solvate and from GSF Form I is presented in Figures S4 and S5.

**Table 1:** Solubility of GSF in MeOH, nBuAc, and ACN at 283 K

solvent	solid form	C* (mg_solute_/g_solvent_)	*x*_eq_ (mmol/mol)	reference
MeOH	GSF form I	3.17 ± 0.05	0.29	this work
		3.21	0.29	^77^
nBuAc	GSF form I	3.68 ± 0.06	1.21	this work
		4.28	1.40	^77^
	GSF-nBuAc	5.64 ± 0.07	1.85	this work
ACN	GSF form I	32.02 ± 0.08	3.71	this work
		33.69	3.90	^77^
	GSF-ACN	34.19 ± 0.02	3.96	this work

**Table 2:** Crystallization Parameters for GSF in MeOH, nBuAc, and ACN

GSF in MeOH							
*S* = *x*/*x*_eq_	RT ln *S* (J mol^–1^)	*t*_50_ (10^3^ s)	*t*_g_ (10^3^ s)	*t*_nuc_ (10^3^ s)	CV	*J* (m^–3^ s^–1^)	*t*_g_ (% of *t*_50_)
1.83	1423	15.26	1.00	14.26	0.88	3.28	6.5
1.90	1523	10.78	0.50	10.28	0.98	4.64	4.6
1.98	1608	4.91	0.55	4.36	1.00	10.18	11.3
2.06	1701	2.96	0.34	2.62	1.01	16.92	11.3
2.14	1791	1.91	0.24	1.67	1.05	26.21	12.4

**GSF in nBuAc**							
*S* = *x*/*x*_eq_	RT ln *S* (J mol^–1^)	*t*_50_ (10^3^ s)	*t*_g_ (10^3^ s)	*t*_nuc_ (10^3^ s)	CV	*J* (m^–3^ s^–1^)	*t*_g_ (% of t_50_)
1.62	1136	21.42	2.77	18.65	0.52	2.33	12.9
1.74	1304	15.08	1.35	13.74	0.55	3.31	8.9
1.78	1357	6.16	1.17	4.99	0.95	8.12	18.9
1.86	1461	5.37	0.65	4.72	0.85	9.32	12.0
1.95	1572	2.61	0.32	2.29	0.75	19.16	12.4

GSF in ACN							
*S* = *x*/*x*_eq_	RT ln *S* (J mol^–1^)	*t*_50_ (10^3^ s)	*t*_g_ (10^3^ s)	*t*_nuc_ (10^3^ s)	CV	*J* (m^–3^ s^–1^)	*t*_g_ (% of t_50_)
1.39	775	11.59	0.64	10.96	0.72	4.31	5.5
1.50	955	6.00	0.24	5.76	0.73	8.34	4.0
1.61	1121	4.11	0.14	3.97	0.98	12.16	3.5
1.70	1249	2.47	0.36	2.12	0.50	20.11	14.6
1.83	1423	1.13	0.14	1.05	0.88	44.13	12.0

### Experimental Nucleation Kinetics Analysis

The induction time (*t*_ind_) probability distributions for GSF in MeOH, nBuAc, and ACN are presented in Figure 2, along with fits of the SNM equation (eq 9).

**Figure 2:**
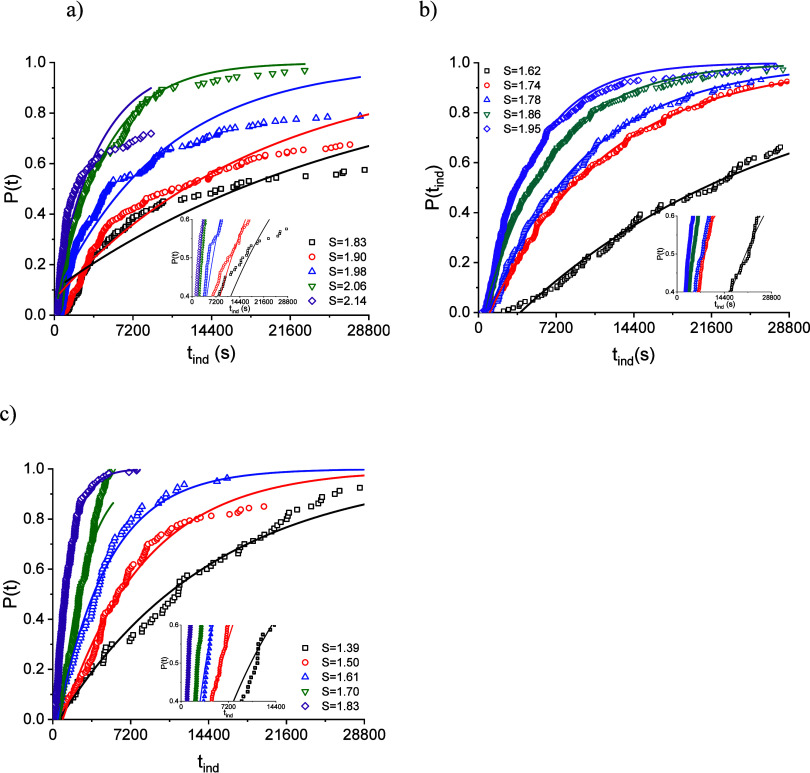
Induction time (*t*_ind_) probability distributions *P*(*t*) at 283 K at different supersaturation ratios (*S*) relative to the nucleation form ranging from (a) 1.83 to 2.14 for GSF in MeOH, (b) 1.62 to 1.95 for GSF in nBuAc, and (c) 1.39 to 1.83 for GSF in ACN. The solid lines show the SNM distribution function fitted to the experimental data. A magnified image of 0.4 ≤ *P*(*t*) ≤ 0.6 is shown as an inset.

Not all the vials nucleated within the time frame analyzed, especially for the lower supersaturations, as shown by probabilities not reaching 1. This is due to an insufficient observation time. The SNM distribution fitted well to the experimental data with coefficients of determination (*R* ^2^) exceeding 0.9 in most cases, except for GSF in MeOH at *S* = 1.83 and *S* = 2.14. The fitted parameters are presented in the Supporting Information (Tables S1–S3). A magnified image of 0.4 ≤ *P*(*t*_ind_) ≤ 0.6 is shown in each figure. It is important to note that the free base nucleates in MeOH, whereas solvated forms nucleate in ACN and nBuAc. Given that the solubility of GSF as Form I is lower than that of its solvated forms in ACN and nBuAc, a hypothetical experiment conducted at the same solution concentration and nucleation temperature where the free base nucleates would result in a higher supersaturation. Consequently, the probability distribution for GSF nucleating as Form I in the measured region would be shifted to the right compared to that of GSF nucleating as solvated forms.

In MeOH, the first nucleation run took longer than the subsequent three runs within the same set of vials, as shown in Figure S6a. This tendency was reproducible between two independent sets of 80 experiments, as shown in Figure S6b. GSF nucleating in nBuAc showed good reproducibility between the four runs, Figure S7a, but showed divergence among five independent sets of 80 experiments, Figure S7b. GSF nucleation in ACN showed a good reproducibility between the four runs of the same solution and between two fresh solutions, Figure S8. Therefore, at least two fresh solutions in each concentration were prepared in MeOH and nBuAc to overcome the higher stochasticity, but one fresh solution was deemed sufficient in ACN.

The median induction time, growth time, nucleation time, coefficient of variation, nucleation rate calculated using the median induction time and volume (*J* = 1/(*t*_50_ *V*)), and growth time as a percentage of median induction time are all presented in Table 2 for GSF in MeOH, nBuAc, and ACN. The growth time was found to be less than 13% of the median induction time for GSF in MeOH, less than 19% for GSF in nBuAc, and less than 15% for GSF in ACN, Table 2. Overall, the median induction time and growth time decreased as S increased, with the exception of the growth time in ACN, where no clear correlation between these parameters was observed with increasing S. The CV can be used as a validation criterion for induction times, tending toward 1 in small volumes and toward 0 in larger volumes.^76^ The CV for the induction time distribution remained within this range, Table 2.

The nucleation rates for GSF are in the range of 3.28–26.21 m^–3^ s^–1^ in MeOH; 2.33–19.16 m^–3^ s^–1^ in nBuAc; and 4.31–44.13 m^–3^ s^–1^ in ACN, at the supersaturation ranges studied; Table 2. These values were of the same magnitude as the nucleation of other organic substances evaluated by classical nucleation theory, which was expected as the supersaturation ranges were selected in order to have nucleation within a reasonable time frame.^12,18,28^ At the same driving force (*S* = 1.83), J is the highest for GSF nucleating in ACN, followed by in nBuAc, and the lowest in MeOH. Thus, nucleation of GSF is the least obstructed in ACN, intermediate in nBuAc, and more difficult in MeOH.

### Classical Nucleation Theory Analysis

The CNT plot with nucleation rate (ln *J*) versus supersaturation ratio (ln^–2^ *S*) is presented in Figure 3. The fitting parameters to the CNT equation are presented in Table S4. The interfacial energy (γ) and the pre-exponential factor (*A*) can be determined by the slope and interception of the CNT plot with the *y*-axis. The interfacial energy for GSF was 3.63 ± 0.12, 2.42 ± 0.18, and 1.69 ± 0.12 mJ m^–2^ in MeOH, nBuAc, and ACN, respectively, Figure 4a. The pre-exponential factors were 1013 ± 491.9, 142.3 ± 101.8, and 68.04 ± 25.95 m^–3^ s^–1^ in MeOH, nBuAc, and ACN, respectively, Figure 4b. For a supersaturation ratio of *S* = 1.83, the nucleation work for GSF, calculated by eq 4, is 5.89, 2.75, and 0.34 *k*_B_ *T* in MeOH, nBuAc, and ACN, respectively. This agrees with the order of nucleation rate: the higher the energy barrier for nucleation, the slower the nucleation rate.^79^ The critical nucleus radius, calculated by eq 5, at *S* = 1.83 is 1.23 ± 0.04, 1.03 ± 0.05, and 0.69 ± 0.07 nm in MeOH, nBuAc, and ACN, respectively (or in terms of the critical diameter, 2.46 ± 0.08, 2.06 ± 0.10, and 1.38 ± 0.14 nm in MeOH, nBuAc, and ACN, respectively). This is consistent with the ease of nucleation: a smaller critical nucleus size facilitates random molecule assembly to produce a stable nucleus.^55^ The number of GSF molecules in the critical nucleus, calculated by eq 6, is 19, 9, and 3 in MeOH, nBuAc, and ACN, respectively. This agrees with reported numbers of organic molecules making up the critical nucleus in the literature, which can range from 1 to a few hundred.^19,39^ It is important to reiterate that if GSF nucleated as Form I in ACN and nBuAc, the probability distribution of *t*_ind_ would shift to the right, indicating a longer induction time and thus a lower nucleation rate in these solvents compared to MeOH.

**Figure 3:**
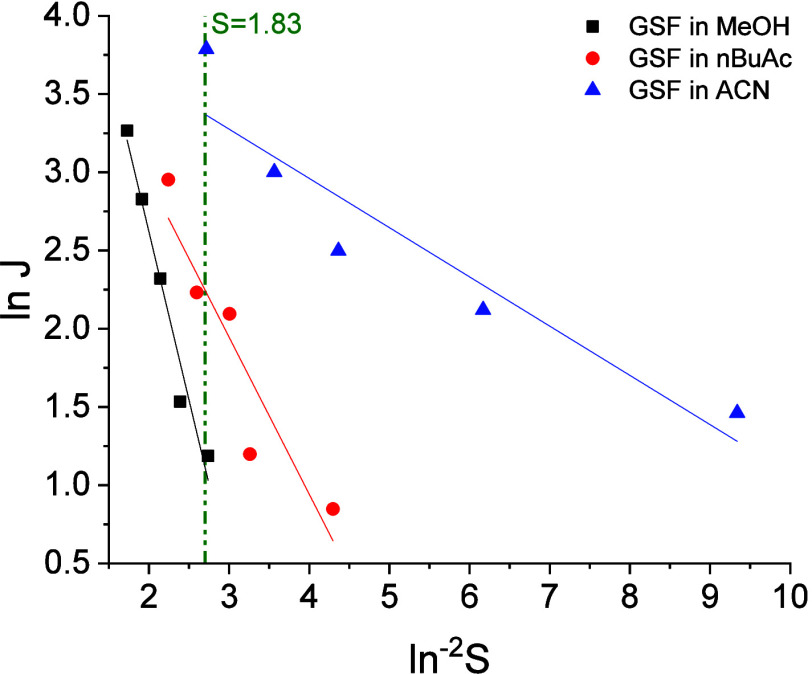
Relationship between nucleation rate (ln *J*) and supersaturation ratio (ln^–2^ *S*) for GSF nucleated at 283 K in MeOH, nBuAc, and ACN. The straight lines are the fitting with the CNT equation (eq 7) and the dotted vertical line corresponds to *S* = 1.83.

**Figure 4:**
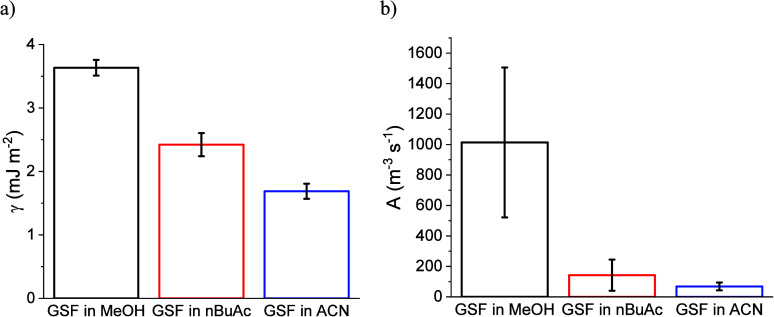
(a) Interfacial energies and (b) pre-exponential factors obtained from CNT plot for GSF nucleating in MeOH, nBuAc, and ACN. Error bars denote standard deviations calculated from the fitting of the CNT plot.

The interfacial energy increases with decreasing solubility across the solvents (Table 2), as can be seen in Figure 5b. The order of interfacial energies is in agreement with what has been proposed by Mersmann (1990), in that interfacial energy decreases with increasing solubility.^77,79^ This has also been observed experimentally for flufenamic acid,^25^ fenoxycarb,^10^ tolbutamide,^29^ etoricoxib,^17^ tolfenamic acid,^38^ and *p*-nitrobenzoic acid.^16^ The nucleation rate increases with increasing solubility (Figure 5c) and decreasing interfacial energy, which has also been observed experimentally for some APIs.^10,17,29,38,39^ However, contrary to expectation, the pre-exponential factor was found to decrease with increasing nucleation rate, being higher in MeOH and similar in ACN and nBuAc, and not showing a correlation to solubility or ease of nucleation (Figure 5a). This trend is also inconsistent with the value of the solubility to viscosity ratio in the solutions,^14^ due to the different nucleating forms. This could indicate that the effect of the solvent in the crystallization of GSF is more affected by solute–solvent interactions (γ), such as hydrogen-bonding and solvation, than by the attachment frequency (*A*). Furthermore, the pre-exponential factor increases with increasing interfacial energy, Figure 5d, which has also been previously reported for salicylamide.^14,32^ This can indicate the presence of a compensating factor, which was explained by Shiau et al. as the solute–solvent interaction leading to a higher interfacial energy, γ. Consequently, at higher γ, the nucleation rate *J* is inhibited, leading to the requirement of a higher rate of attachment, i.e., a higher A, to achieve the same nucleation rate.^32^

**Figure 5:**
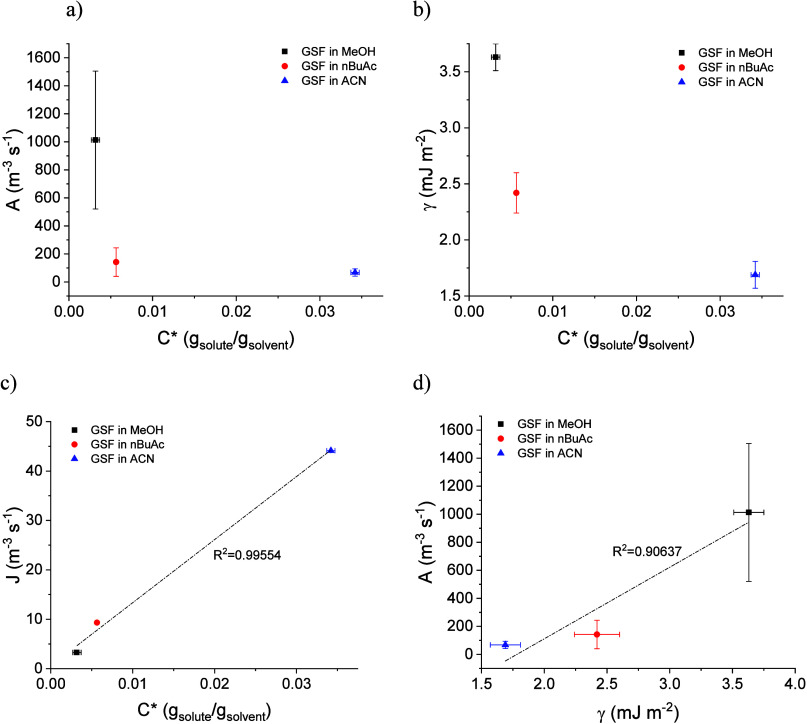
Relationship between (a) pre-exponential factor, (b) interfacial energy, and (c) nucleation rate at *S* = 1.83 with the solubility at 283 K and (d) pre-exponential factor with interfacial energy.

Alternative methods to extract nucleation parameters from the experimental data by considering the growth time effects as being negligible (method 1) or not (method 2) for the nucleation time and by extracting the parameters from the fitting with the SNM mechanism (method 3) were explored (Figure S9). Although these methods provided different quantitative results from the ones determined through the CNT, they followed the same trend and therefore provided similar qualitative results, as shown in Figure S10. This suggests that the growth time is insignificant compared to the median induction time and that the SNM provides a reliable determination of the nucleation parameters.

### Mesoscale Cluster Analysis

The presence of mesoscale clusters in GSF solutions was investigated at a supersaturation of 1.83, following thermal pretreatment similar to the conditions used in the nucleation experiments. Two decays were observed on the correlogram for ACN and nBuAc, whereas no second decay was observed for MeOH, as shown in Figure 6. This behavior was observed in all four replicates analyzed for the samples (Figure S11). A correlogram with two decays is typical of solutions containing mesoscale clusters and has been previously reported for aqueous solutions of glycine,^46,48,49^ dl-alanine,^49^ and ethanolic solutions of carbamazepine-saccharin.^80^ No scattering species were detected in blank solvents (Figure S13).

**Figure 6:**
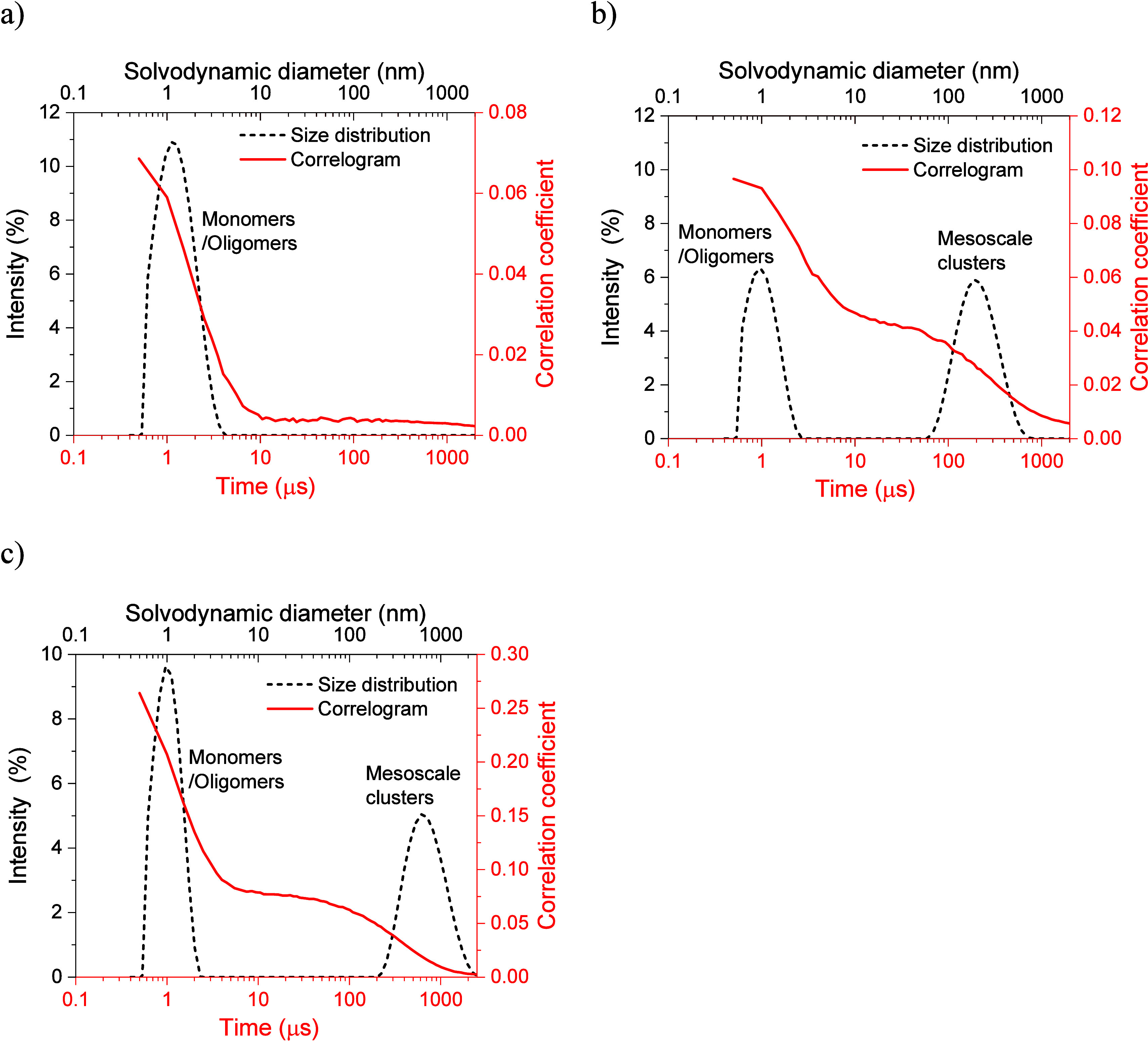
Overlay of correlogram with corresponding size distribution at *S* = 1.83 for GSF in (a) MeOH, (b) nBuAc, and (c) ACN.

In the case of GSF in ACN (Figure 6c), the initial decay was relatively fast, with a characteristic decay time of less than 0.007 ms. The average solvodynamic diameter (*D*_s_) correlating to this peak is 1.01 ± 0.08 nm, corresponding to the dissolved GSF and could be from solvated GSF and the corresponding oligomers. The second decay was relatively slow with a characteristic decay time of less than 3 ms, indicating an average mesoscale cluster diameter of 620 ± 335 nm. Similarly, for GSF in nBuAc (Figure 6b), the characteristic decay time for the first decay was less than 0.012 ms, while the second decay had an average decay time of less than 2 ms. In this solvent, the monomer/oligomer peak has an average *D*_s_ of 1.12 ± 0.04 nm, while the average mesoscale diameter is 176 ± 57.0 nm. Finally, in the case of GSF in MeOH (Figure 6a), where only one decay was observed, the characteristic decay times were less than 0.010 ms, leading to a monomer/oligomer peak of 1.40 ± 0.01 nm and no mesoscale cluster peak.

It has been observed that the presence of clusters in solutions often leads to faster nucleation as compared to solutions without clusters.^46^ Furthermore, it has been shown that larger cluster sizes or higher cluster number concentrations lead to higher nucleation rates.^45,55,56^ Mesoscale clusters were detected in solutions of ACN and nBuAc, while no mesoscale clusters were detected for GSF in MeOH at the same supersaturation level, temperature, and pretreatment. This suggests that mesoscale clusters could be responsible for the faster nucleation observed in ACN and nBuAc. Additionally, as shown in Figure 6, the y-intercept corresponds to the correlation coefficient and represents the scattered intensity reaching the detector. A higher scattered intensity could be attributed to factors such as higher cluster size, higher cluster number concentration, or higher cluster mass density. The intensity of scattering was highest in ACN, intermediate in nBuAc, and lowest in MeOH, which corresponds well with the order of nucleation rate.

The derived count rate (DCR) represents the average scattering intensity and is an indicator of cluster number concentration, size, and/or mass density.^72^ At the same supersaturation level (*S* = 1.83), the mesoscale cluster size (*D*_s_) and concentration (DCR) for GSF-ACN (620 ± 335 nm and 737 ± 124 kcps) tend to be higher than for GSF-nBuAc (176 ± 57.0 nm and 340 ± 63.6 kcps), Figure 7.

**Figure 7:**
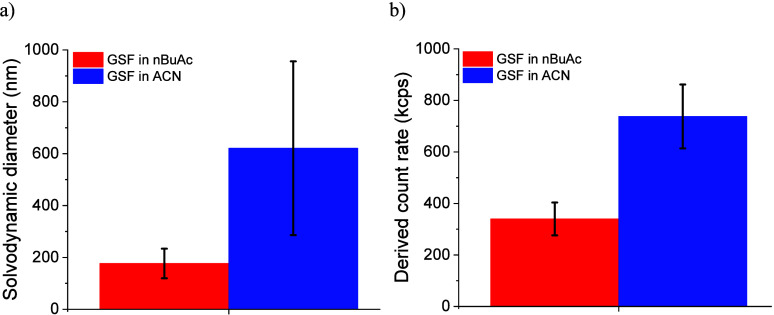
Comparison of (a) solvodynamic diameter and (b) derived count rate for GSF clusters in nBuAc and ACN at *S* = 1.83 from dynamic light scattering analysis. Number of samples = 4.

In the literature, nonclassical nucleation has been reported to occur via mesoscale clusters for organic molecules.^40^ The results of the current study indicate that, in the case of GSF in ACN and nBuAc, the free energy of the mesoscale clusters (*G*_cluster_) might be lower than that of the solution (*G*_solution_), resulting in the spontaneous formation of stable mesoscale clusters upon solid dissolution.^40,46^ On the other hand, for GSF in MeOH, where mesoscale clusters were not detected at the onset of nucleation, this could indicate that the free energy of the mesoscale clusters (*G*_cluster_) might be higher than that of the solution (*G*_solution_), resulting in metastable mesoscale clusters that are not spontaneously formed upon solid dissolution but can still be formed during the prenucleation phase.^40,81^ The solvodynamic mesoscale cluster diameters, 176 ± 57 nm in nBuAc and 620 ± 335 nm in ACN, were larger than the critical nucleus diameter calculated by CNT, 2.06 ± 0.10 nm in BuAc and 1.38 ± 0.15 nm in ACN, or the molecular diameter calculated by the crystal structure molecular volume, 0.99 nm in nBuAc and 0.97 nm in ACN; Table S6. This is due to the assumption in CNT that the nuclei formed are composed only of solute molecules (or the final stoichiometric ratio of solute:solvate in the nucleating form). In nonclassical nucleation theories, the clusters formed are assumed to contain both solute and solvent molecules, and, depending on the theory, the crystalline nucleus then forms within the cluster over time. Thus, it is to be expected that the critical nucleus diameter determined by CNT is smaller than the measured mesoscale cluster diameter that is formed in the nonclassical nucleation pathway for crystallization from solution.

In the present study, the critical nucleus size determined by CNT at the same supersaturation level (*S* = 1.83) is smaller in ACN, intermediate in nBuAc, and higher in MeOH. This aligns with the reported correlation as larger cluster sizes were observed in ACN, followed by nBuAc, with no clusters detected in MeOH. If critical nuclei form within these clusters, it would be logical to assume that nucleation is easiest in solutions with larger cluster sizes/densities, such as ACN, followed by nBuAc with smaller clusters. In the case of GSF in MeOH, where no clusters are present, nucleation would be the most difficult. This trend is also consistent with the order of interfacial energy, which measures the difficulty of forming a new phase in solution, being the highest for MeOH, followed by nBuAc, and the smallest in ACN. Thus, it can be hypothesized that the differences in mesoscale cluster properties explain the higher nucleation rate observed in ACN as compared to nBuAc and MeOH.

### Overall Analysis of Nucleation

An overall analysis of the different aspects affecting GSF nucleation investigated in this work is presented in Table 3. The ease of nucleation determined experimentally correlates well with the interfacial energy determined by the CNT equation (eq 7): faster nucleation corresponds to a lower γ. However, the ease of nucleation does not correlate well with the pre-exponential factor A, which is found to be higher in MeOH, where nucleation is slower. It could be argued that the impact of the higher pre-exponential factor on nucleation rate is negated by the higher interfacial energy in MeOH. Therefore, we hypothesize that the interfacial energy determined by CNT adequately describes the effect of solvent on the nucleation kinetics of GSF among the evaluated solvents. However, the kinetic factor (*A*) did not correlate with the CNT analysis, as has been previously reported for other systems,^9–11,14,17,18,27,29,38,39^ indicating there might be an alternative mechanism of nucleation at play. This led to the observation of faster nucleation in solutions in which mesoscale clusters were detected (nBuAc and ACN), particularly with larger sizes and higher concentrations (ACN). Hence, analyzing the presence of mesoscale clusters may offer a more comprehensive understanding of the underlying mechanism of nucleation, as presented in this work.

**Table 3:** Correlation of Nucleation Parameters and Solution Properties between Experimental Data and Classical and Nonclassical Analysis of Nucleation of Griseofulvin in MeOH, nBuAc, and ACN

	experimental nucleation at *S* = 1.83		classical nucleation theory analysis			nonclassical nucleation theory analysis at *S* = 1.83		
solvent	*t*_nuc_ (s)	*J* (m^–3^s^–1^)	γ (mJ m^–2^)	*A* (m^–3^s^–1^)	*D*_c_ (nm) at S–1.83	*D*_s_ (monomer) (nm)	*D*_s_ (cluster) (nm)	DCR (kcps)
MeOH	14261	3.28	3.63 ± 0.12	(1.01 ± 0.49) × 10^3^	2.46 ± 0.08	1.40 ± 0.01		
nBuAc	4720	9.32	2.42 ± 0.18	(1.42 ± 1.01) × 10^2^	2.06 ± 0.10	1.12 ± 0.04	176 ± 57.0	340 ± 63.6
ACN	1047	44.1	1.69 ± 0.12	(6.80 ± 2.59) × 10^1^	1.38 ± 0.14	1.01 ± 0.08	620 ± 335	737 ± 124

A nucleation pathway via mesoscale clusters could involve initial formation/aggregation/growth of mesoscale clusters, followed by nucleation within these solute-rich regions.^40^ A summary of the GSF nucleation results in different solvents is presented in Figure 8. In the case of GSF in ACN, where clusters are already present, these clusters may serve as nucleation sites or further aggregate and grow under agitation. Both the pre-existing clusters and their aggregated forms can potentially act as nucleation sites. Similarly, for GSF in nBuAc, a comparable pathway is plausible, as clusters are present at the onset of nucleation (Figure 8). In the case of MeOH, although no clusters were detected under the resolution limit of DLS, clusters may still form during the incubation process and subsequently aggregate or serve as nucleation sites. However, the possibility that GSF in MeOH may nucleate via a classical pathway cannot be ruled out. Furthermore, it is noteworthy that in the solutions where clusters were detected, GSF nucleated as solvated forms (Figure 8).

**Figure 8:**
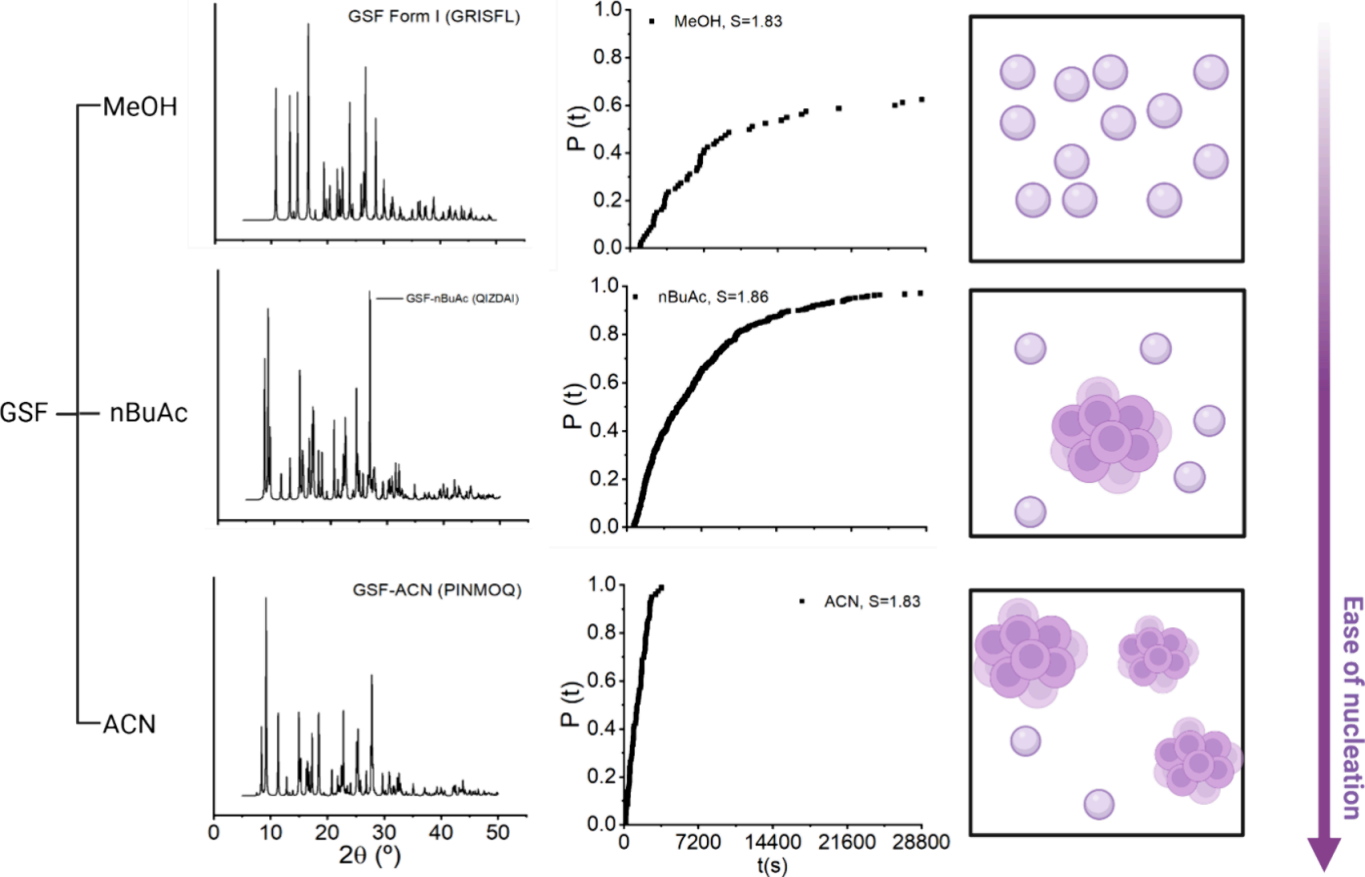
Overall summary of GSF nucleation in different solvents.

## Conclusions

In this work, we present an evaluation of the effect of solvent on the nucleation of GSF through classical and nonclassical mechanisms. The nucleation kinetics and resulting solid form were observed to be solvent-dependent since GSF exhibits a higher nucleation rate as a solvated form in both acetonitrile (ACN) and *n*-butyl acetate (nBuAc), whereas in methanol (MeOH), it presents a lower nucleation rate, favoring the formation of the thermodynamically stable Form I. The ease of nucleation at a given supersaturation level correlated well with the thermodynamic parameter of the CNT equation, the interfacial energy, and the overall work required for nucleation. In ACN and nBuAc, GSF mesoscale clusters were detected in solution, suggesting that nucleation may follow a nonclassical nucleation pathway in these solvents. GSF nucleation was faster in ACN, which had a higher concentration and larger size of mesoscale clusters compared to that in nBuAc at an equivalent supersaturation level. In MeOH, GSF exists predominantly as monomers/dimers in solution, resulting in slower nucleation than that in ACN and nBuAc. This suggests nucleation through a classical pathway or, if a nonclassical pathway is followed, with metastable mesoscale clusters that need to form during the prenucleation phase. This work highlights the complexity of nucleation pathways and the necessity of further investigation into the impact of the solvent choice to better understand this process.

## Supplementary Material



## Notes

Powder X-ray diffraction patterns of griseofulvin crystallized from the systems analyzed, powder X-ray diffraction patterns of the griseofulvin solid after equilibration for solubility measurements, induction time distributions for GSF in MEOH, nBuAc, and ACN, parameters for the fitting of the experimental nucleation time distribution to the theoretical single nucleus mechanism, parameters for the fitting of classical nucleation theory plot, methods for calculating pre-exponential factor (*A*) and interfacial energy (γ) from the nucleation rate (*J*), calculation of the uncertainty, mesoscale cluster analysis, and analysis of size of critical nucleus, monomer, and the molecular crystal structure (PDF)

## Abbreviations

*A*: Pre-exponential factor
ACN: Acetonitrile
*C**: Equilibrium solubility
CSD: Cambridge structural database
CV: Coefficient of variation
GSF: Griseofulvin
*J*: Nucleation rate
*k*_B_: Boltzmann constant
MW: Molecular weight
MeOH: Methanol
*n*: Number of nuclei formed during a time interval
*N*: Average number of nuclei formed during a time interval
nBuAc: *n*-butyl acetate
*P*: Probability distribution
PXRD: Powder X-ray diffraction
*r**: Critical radius
*S*: Supersaturation ratio
*T*: Absolute temperature
*t*_g_: Growth time
*t*_ind_: Induction time
*t*_nuc_: Nucleation time
*V*: Solution volume
*x*: Mole fraction
γ: Surface energy
Δ*G**: Critical free energy
υ: Molecular volume
