# Supplementary material for: Rationalizing the Influence of Solvent on the Nucleation of Griseofulvin through Classical and Nonclassical Pathways
Source: Cryst Growth Des. 2025 Jun 3;25(13):4713–24. doi: 10.1021/acs.cgd.5c00206 (PMC12232334; doi:10.1021/acs.cgd.5c00206)
Supplement: Supplementary file 1 [file cg5c00206_si_001.pdf]

Supporting information

RATIONALISING THE INFLUENCE OF SOLVENT ON THE NUCLEATION OF GRISEOFULVIN THROUGH CLASSICAL AND NON-CLASSICAL PATHWAYS

Mariana O. Diniz[1], Harsh Barua[1], Jennifer Cookman[1], Michael Svärd[2], Åke Rasmuson[1,2], Sarah P. Hudson[1]*

[1] SSPC the Research Ireland Centre for Pharmaceuticals, Department of Chemical Sciences, and Bernal Institute, University of Limerick, Ireland.

[2] Department of Chemical Engineering, KTH Royal Institute of Technology, Stockholm, Sweden.

TABLE OF CONTENTS

POWDER X-RAY DIFFRACTION OF GRISEOFULVIN CRYSTALLIZED FROM THE SYSTEMS ANALYZED 3

POWDER X-RAY DIFFRACTION OF GRISEOFULVIN SOLID PRESENT AFTER EQUILIBRATION FOR SOLUBILITY MEASUREMENT 4

INDUCTION TIME MEASUREMENTS FOR GSF IN MEOH, nBuAc, AND ACN 6

PARAMETERS FOR THE FITTING OF THE EXPERIMENTAL NUCLEATION TIME DISTRIBUTION TO THE THEORETICAL SINGLE NUCLEUS MECHANISM 7

PARAMETERS FOR THE FITTING OF CLASSICAL NUCLEATION THEORY PLOT ... 8

METHODS FOR CALCULATING PRE-EXPONENTIAL FACTOR (A) AND INTERFACIAL ENERGY (γ) FROM THE NUCLEATION RATE (J) 8

CALCULATION OF THE UNCERTAINTY 10

MESOSCALE CLUSTER ANALYSIS 12

ANALYSIS OF SIZE OF CRITICAL NUCLEUS, MONOMER, AND MOLECULAR CRYSTAL STRUCTURE 14

POWDER X-RAY DIFFRACTION OF GRISEOFULVIN CRYSTALLIZED FROM THE SYSTEMS ANALYZED

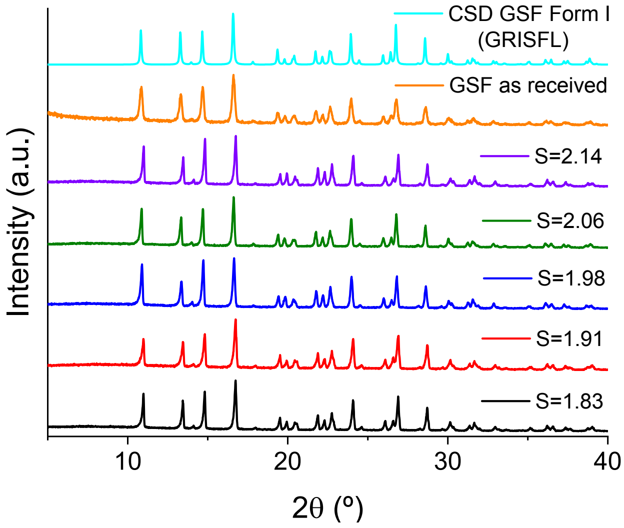

Figure S1. *Powder X-ray diffraction patterns of GSF nucleated in MeOH at different supersaturations, the GSF as received, and the CSD for GSF form I.*

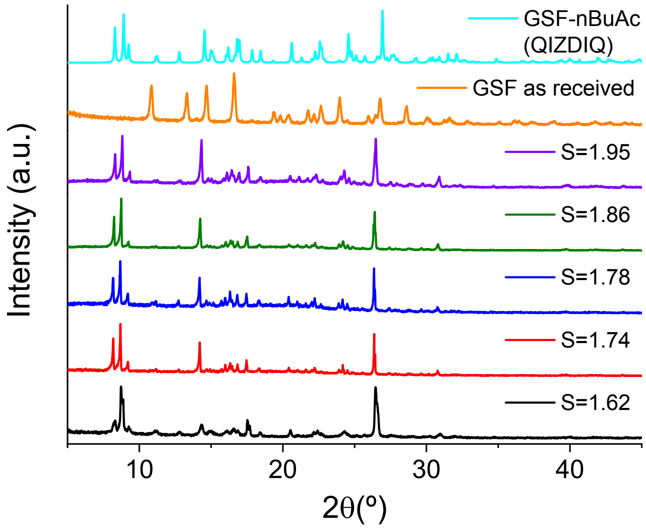

Figure S2. *Powder X-ray diffraction patterns of GSF nucleated in nBuAc freshly filtered at different supersaturations, the GSF as received and the CSD for the GSF-nBuAc solvated form.*

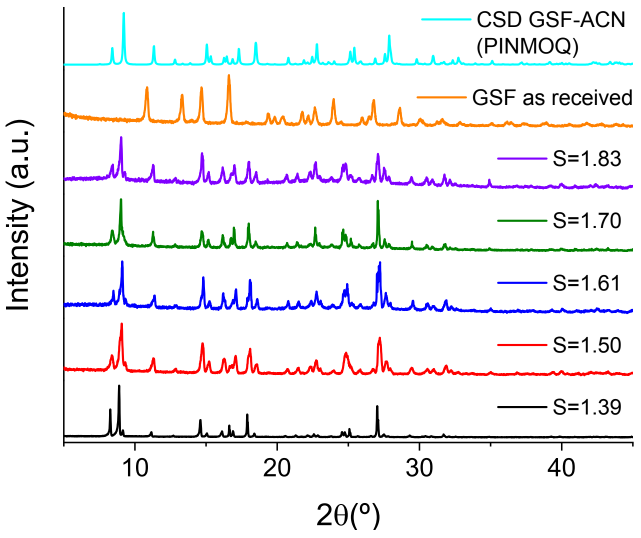

Figure S3. *Powder X-ray diffraction patterns of the slurry of GSF nucleated in ACN at different supersaturations, the GSF as received and the CSD for the GSF-ACN solvated form.*

POWDER X-RAY DIFFRACTION OF GRISEOFULVIN SOLID PRESENT AFTER EQUILIBRATION FOR SOLUBILITY MEASUREMENT

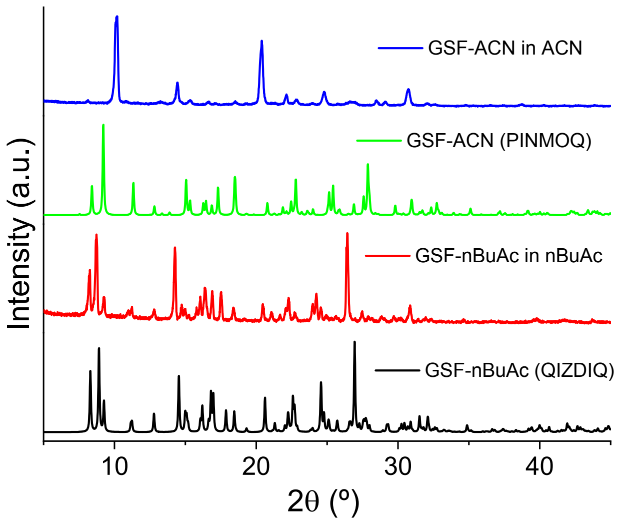

Figure S4. *Powder X-ray diffraction patterns of the slurry of GSF after equilibration for solubility measurement starting with the solvate slurry after nucleation experiments.*

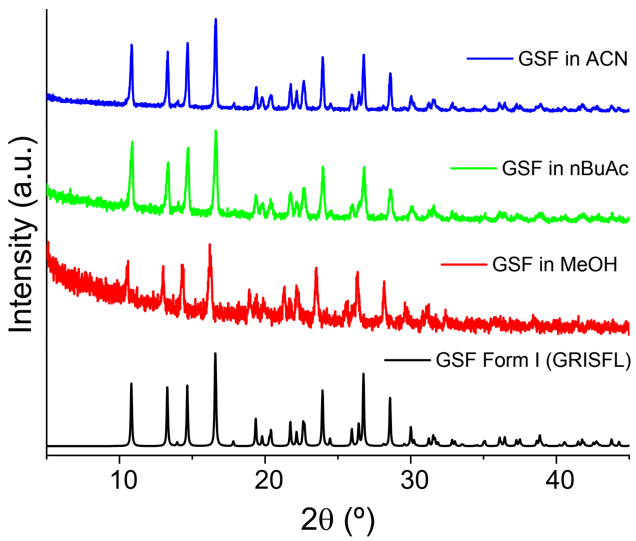

Figure S5. Powder X-ray diffraction patterns of the slurry of GSF after equilibration for solubility measurement starting from GSF form I.

INDUCTION TIME MEASUREMENTS FOR GSF IN MEOH, nBuAc, AND ACN

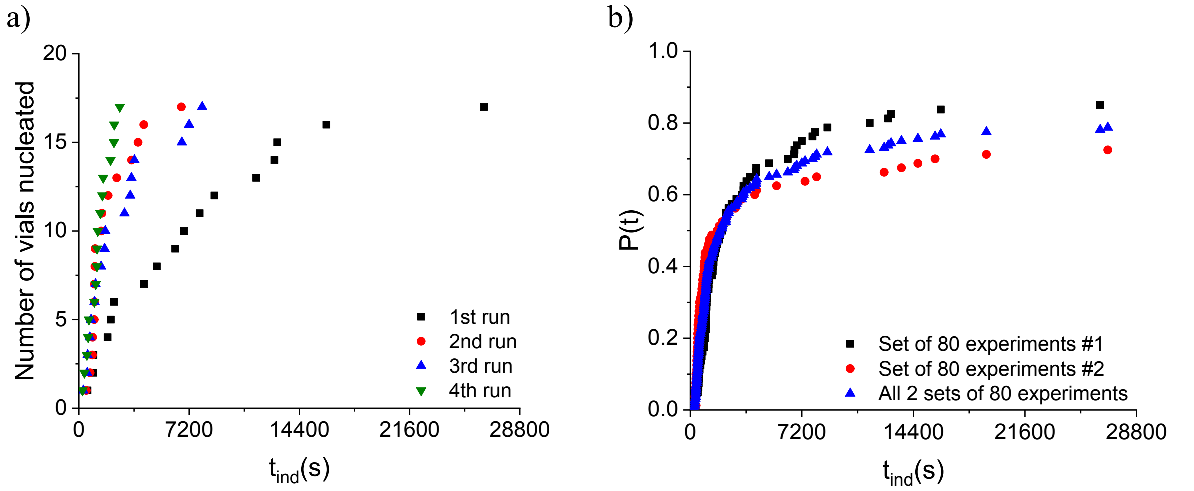

Figure S6. Induction time distribution for GSF in MeOH at a supersaturation level S=2.14 a) between runs within a set of 80 vials, b) between 2 independent sets of 80 vials.

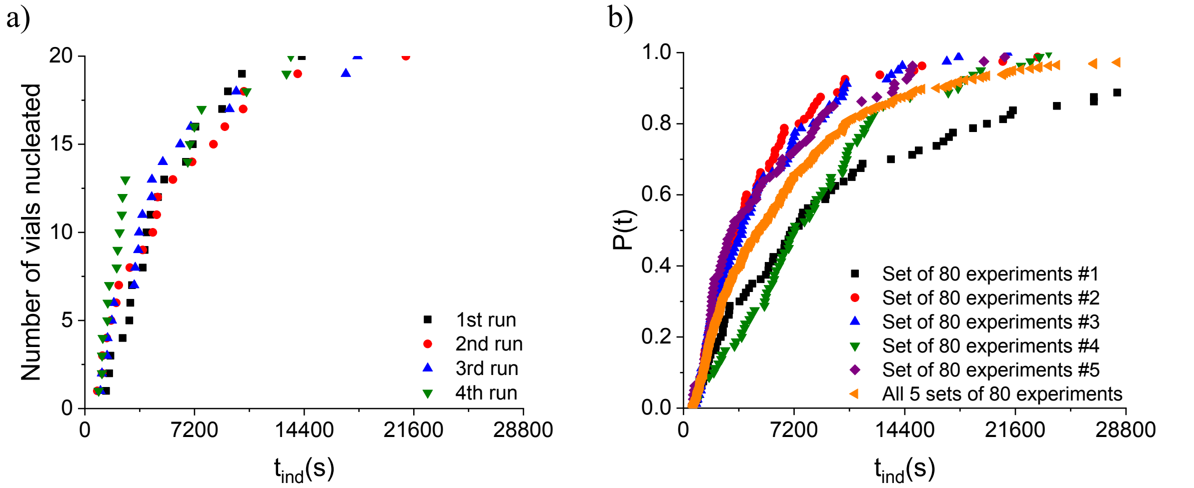

Figure S7. Induction time distribution for GSF in nBuAc at a supersaturation level S=1.86 a) between runs within a set of 80 vials, b) between 5 independent sets of 80 vials.

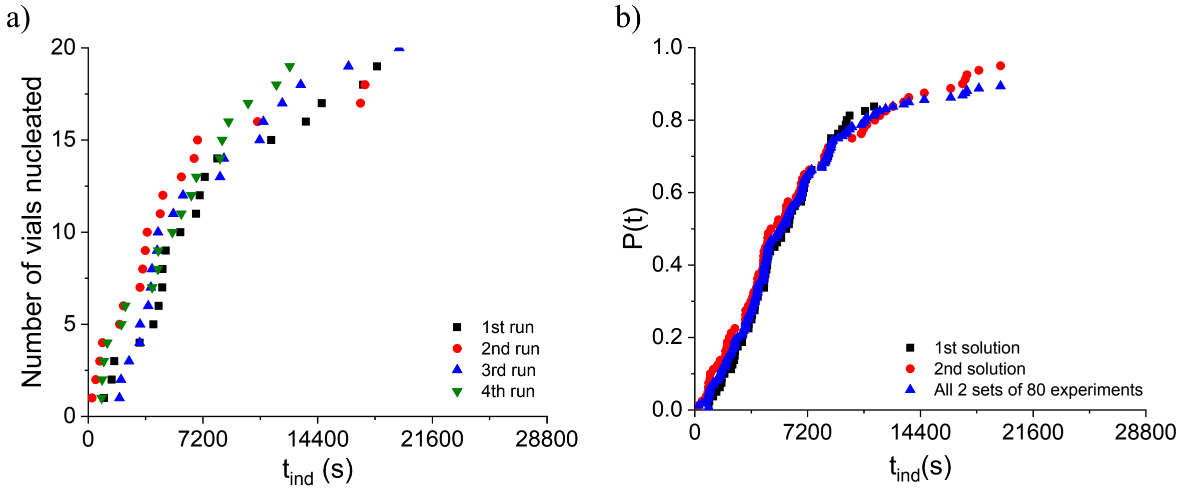

Figure S8. Induction time distribution for GSF in ACN at a supersaturation level S=1.51 a) between runs within a set of 80 vials, b) between 2 independent sets of 80 vials.

PARAMETERS FOR THE FITTING OF THE EXPERIMENTAL NUCLEATION TIME DISTRIBUTION TO THE THEORETICAL SINGLE NUCLEUS MECHANISM

Table S1. Single Nucleus Mechanism fitting parameters to the experimental induction time for GSF in MeOH at 283 K and different supersaturations.

**Model**	**SNM (User)**				
**Equation**	1-exp(-J*V*(t-tg))				
**Plot**	S=1.83	S=1.91	S=1.98	S=2.06	S=2.14
**t_g (s)**	-3039±704	-1217±227	-705±113	22.9±3.2	-297±80
**J (m^{-3} s^{-1})**	1.739±0.128	2.642±0.083	4.974±0.135	11.200±0.177	12.530±0.650
**R^2 (COD)**	0.749	0.907	0.930	0.985	0.856

Table S2. Single Nucleus Mechanism fitting parameters to the experimental induction time for GSF in ACN at 283 K and different supersaturations.

**Model**	**SNM (User)**				
**Equation**	1-exp(-J*V*(t-tg))				
**Plot**	S=1.39	S=1.50	S=1.61	S=1.70	S=1.83
**t_g (s)**	593.6±135.6	591.2±57.6	451.3±42.3	739.1±47.2	165.4±17.9
**J (m^{-3} s^{-1})**	3.466±0.066	7.329±0.128	10.471±0.188	19.571±0.679	36.999±0.960
**R^2 (COD)**	0.984	0.988	0.987	0.941	0.970

Table S3. Single Nucleus Mechanism fitting parameters to the experimental induction time for GSF in nBuAc at 283 K and different supersaturations.

**Model**	**SNM (User)**				
**Equation**	1-exp(-J*V*(t-tg))				
**Plot**	S=1.62	S=1.74	S=1.78	S=1.86	S=1.95
**t_g (s)**	3853.5±119.9	1035.4±17.2	939.9±30.3	507.6±11.5	501.0±13.1
**J (m^{-3} s^{-1})**	2.030±0.024	4.630±0.158	5.472±0.039	7.895±0.031	10.840±0.067
**R^2 (COD)**	0.987	0.999	0.996	0.997	0.994

PARAMETERS FOR THE FITTING OF CLASSICAL NUCLEATION THEORY PLOT

Table S4. Parameters of linear fitting of nucleation of GSF in MeOH, ACN and nBuAc at 283 K experimental data to the CNT plot.

**Equation**	**Y=a+bx**		
**Plot**	GSF in MeOH	GSF in ACN	GSF in nBuAc
**Intercept**	7.19 ± 0.54	4.34 ± 0.40	5.10 ± 0.78
**Slope**	-2.23 ± 0.25	-0.32 ± 0.07	-1.01 ± 0.25
**R^2**	0.965	0.880	0.846

METHODS FOR CALCULATING PRE-EXPONENTIAL FACTOR (A) AND INTERFACIAL ENERGY (γ) FROM THE NUCLEATION RATE (J)

- Method 1

This method does not consider the growth time (t_g) affecting the nucleation time (t_{nuc}=t_{50}).

$$J = \frac{1}{t_{50} \times V}$$

- Method 2

This method considers the growth time (t_g) and induction time (t_{50}) affecting the nucleation time (t_{nuc}): $t_{nuc} = t_{50} - t_g$.

$$J = \frac{1}{t_{nuc} \times V}$$

- Method 3

This method considers the fitting with the Single Nucleus Mechanism (SNM) fitting to the experimental induction time distribution.

$$P(t) = 1 - e^{-J \times V (t - t_g)}$$

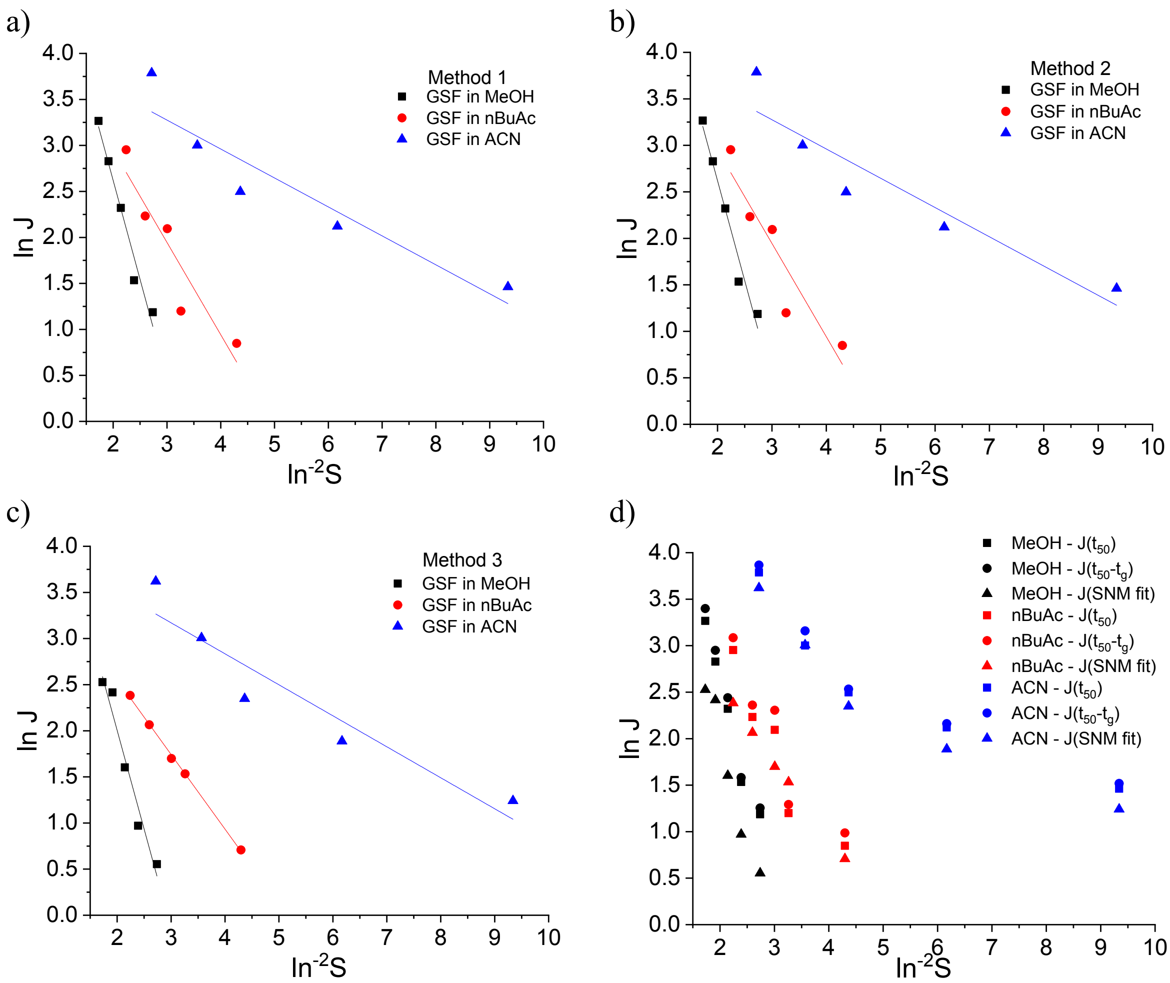

Figure S9. Methods for calculating nucleation parameters for GSF nucleating in MeOH, nBuAc, and ACN.

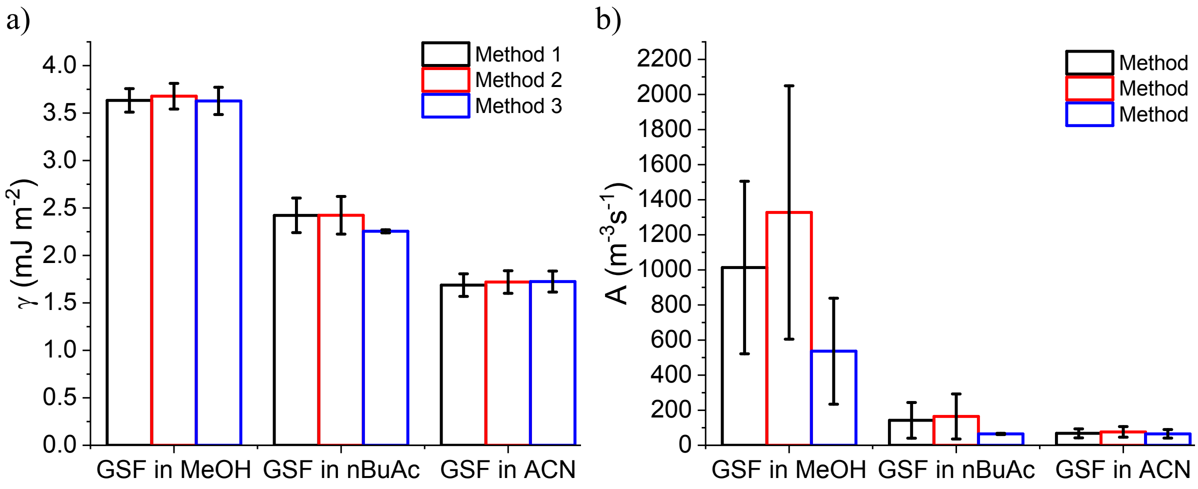

Figure S10. Nucleation parameters calculated from different methods for GSF nucleating in MeOH, nBuAc, and ACN a) pre-exponential factor and b) interfacial energy. The error bars are calculated based on the uncertainty of the CNT fitting equation.

CALCULATION OF THE UNCERTAINTY

The uncertainty was calculated by the error propagation formula. For a function y = f(x), the propagated uncertainty σ_y is given by:

$$\sigma_y = \left|\frac{\partial f}{\partial x}\right| \sigma_x$$

- For the pre-exponential factor:

$$A = f(intercept) = e^{intercept}$$

$$\frac{\partial f}{\partial intercept} = \frac{\partial e^{intercept}}{\partial intercept} = e^{intercept}$$

$$\sigma_A = \frac{\partial f}{\partial intercept} \sigma_{intercept}$$

$$\sigma_A = e^{intercept} \times \sigma_{intercept}$$

$$A = e^{intercept} \pm e^{intercept} \times \sigma_{intercept}$$

- For the interfacial energy:

$$\gamma = f(slope) = \sqrt[3]{\frac{3k^3T^3 slope}{16\pi v^2}}$$

$$\frac{\partial f}{\partial slope} = \frac{1}{3}\left(\frac{3k^3T^3 slope}{16\pi v^2}\right)^{-2/3} \frac{3k^3T^3}{16\pi v^2}$$

Simplifying

$$\frac{\partial f}{\partial slope} = \frac{1}{3}\frac{\gamma^2}{slope}$$

$$\sigma_\gamma = \frac{\partial f}{\partial slope} \sigma_{slope}$$

$$\sigma_\gamma = \frac{1}{3}\frac{\gamma^2}{slope} \times \sigma_{slope}$$

$$\gamma = \gamma \pm \frac{1}{3}\frac{\gamma^2}{slope} \times \sigma_{slope}$$

- For the critical radius:

$$r = f(\gamma) = \frac{2\gamma\upsilon}{KTlnS}$$

$$\frac{\partial f}{\partial \gamma} = \frac{2\upsilon}{KTlnS}$$

$$\sigma_r = \frac{2\upsilon}{KTlnS} \times \sigma_\gamma$$

$$r = \frac{2\gamma\upsilon}{KTlnS} \pm \frac{2\upsilon}{KTlnS} \times \sigma_\gamma$$

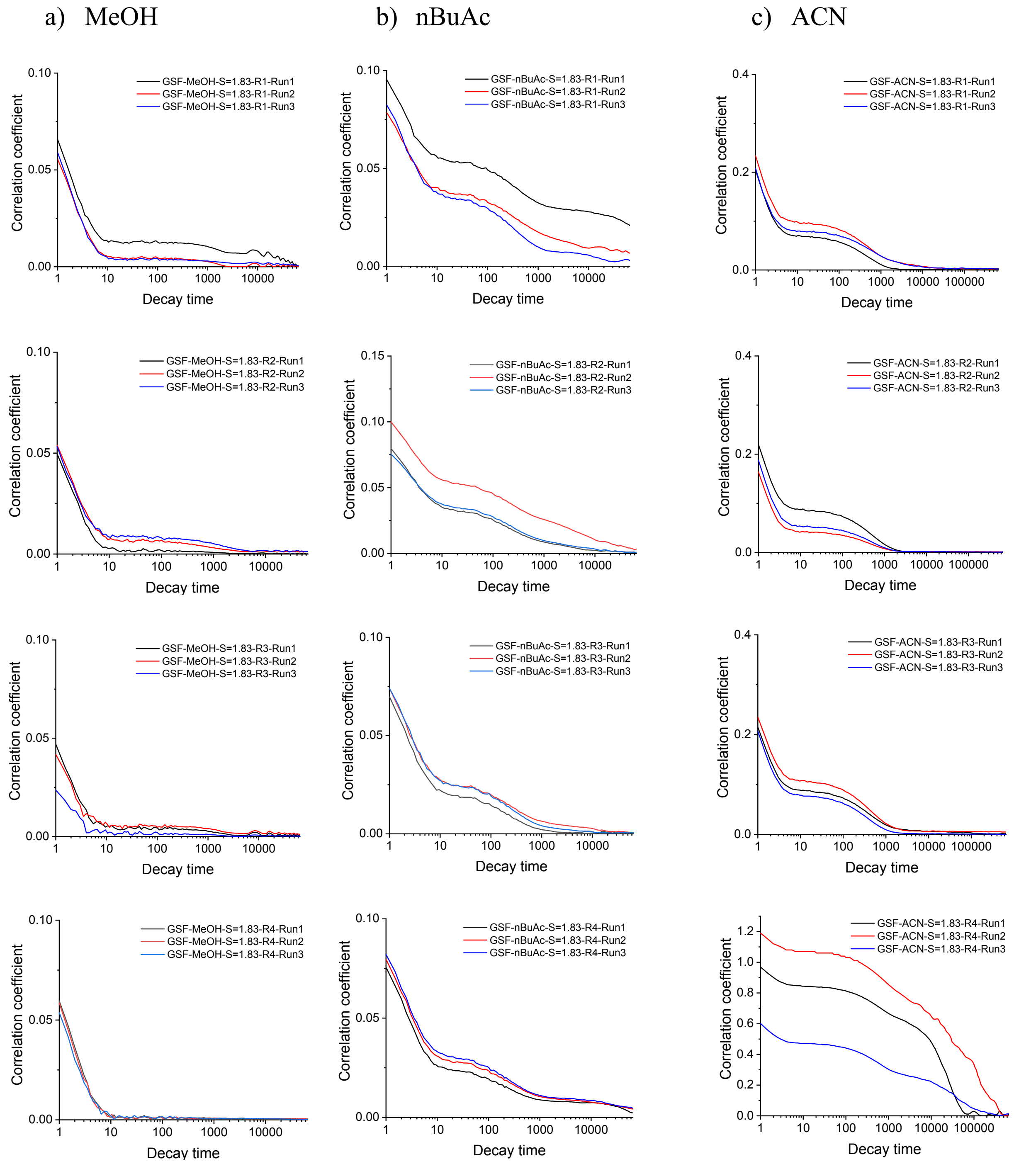

Figure S11. DLS correlogram for GSF in a) MeOH, b) nBuAc and c) ACN at S = 1.83 for all the four replicates.

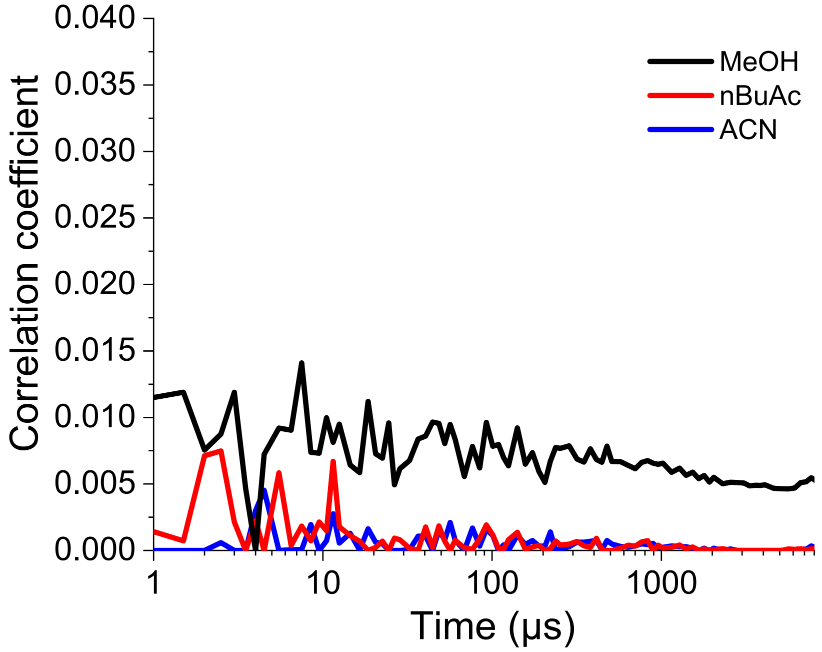

Figure S12. DLS correlogram for pure solvents: a) MeOH, b) nBuAc, and c) ACN.

ANALYSIS OF SIZE OF CRITICAL NUCLEUS, MONOMER, AND MOLECULAR CRYSTAL STRUCTURE

Table S5. Analysis of size of critical nucleus, monomer, and molecular crystal structure.

		MeOH	nBuAc	ACN
CNT	γ (mJ m^{-2})	3.63 ± 0.12	2.42 ± 0.12	1.69 ± 0.18
	Critical nucleus radius (nm)*	1.23 ± 0.04	1.03 ± 0.05	0.69 ± 0.07
	Critical nucleus diameter (nm)*	2.46 ± 0.08	2.06 ± 0.10	1.38 ± 0.15
DLS	Solvent viscosity	0.70[1]	0.85[2]	0.40[1]
	Ds monomer (nm)*	1.4 ± 0.01	1.12 ± 0.04	1.01 ± 0.08
	Ds cluster (nm)*	-	176 ± 57	620 ± 335
CSD	Molecular volume (nm^3)	0.40	0.50	0.48
	Molecular radius (nm)	0.46	0.49	0.49
	Molecular diameter (nm)	0.91	0.99	0.97

Note: * Parameters obtained at S = 1.83, T = 283K.

REFERENCES

1. Nikam, P.S., Shirsat, L.N. and Hasan, M. Density and viscosity studies of binary mixtures of acetonitrile with methanol, ethanol, propan-1-ol, propan-2-ol, butan-1-ol, 2-methylpropan-1-ol, and 2-methylpropan-2-ol at (298.15, 303.15, 308.15, and 313.15) K, J. *Chem. Eng. Data*. **43**, 732-737 (1998).
2. Rathnam, M., Sayed, R.T., Bhanushali, K.R. and Kumar, M. Density and viscosity of binary mixtures of n-butyl acetate with ketones at (298.15, 303.15, 308.15, and 313.15) K, J. *Chem. Eng. Data*. **57**, 1721-1727 (2012).
